# Phytochemical composition and antimicrobial potential of *Stevia rebaudiana* Bertoni extract and its topical spray formulation against animal skin pathogens

**DOI:** 10.14202/vetworld.2024.2975-2984

**Published:** 2024-12-30

**Authors:** Ranee Singh, Glenn Neville Borlace, Patchanee Sringam, Eakachai Thongkham, Jareerat Aiemsaard

**Affiliations:** 1 Division of Pharmacology and Toxicology, Faculty of Veterinary Medicine, Khon Kaen University, Khon Kaen, Thailand; 2 Department of Pharmaceutical Chemistry, Faculty of Pharmaceutical Sciences, Khon Kaen University, Khon Kaen, Thailand; 3 Division of Physiology, Faculty of Veterinary Medicine, Khon Kaen University, Khon Kaen, Thailand; 4 Department of Pharmacology, Faculty of Veterinary Medicine, Mahanakorn University of Technology, Bangkok, Thailand

**Keywords:** animal skin infection, antimicrobial activity, *Stevia rebaudiana*, topical formulation

## Abstract

**Background and Aim:**

The rise of antimicrobial resistance in veterinary medicine is a significant concern, particularly for pathogens responsible for skin infections. Although *Stevia rebaudiana* Bertoni (stevia) has demonstrated effective antimicrobial properties, there is limited research on its efficacy against animal skin pathogens. This study aimed to identify natural compounds in stevia extract, develop a topical spray formulation, and assess its effectiveness against six common bacterial and fungal pathogens associated with animal skin infections.

**Materials and Methods:**

The aerial parts of stevia plants were extracted using hexane in a Soxhlet apparatus. Total phenolic and flavonoid contents were quantified using colorimetric assays. The volatile oil content was analyzed using gas chromatography-mass spectrometry (GC-MS). The antimicrobial activity of stevia extract against *Staphylococcus pseudintermedius*, *Malassezia pachydermatis*, *Microsporum canis*, *Microsporum gypseum*, *Microsporum gallinae*, and *Trichophyton mentagrophytes* was evaluated using broth microdilution and time-kill tests. Environmental scanning electron microscopy (E-SEM) and leakage studies were conducted to assess the extract’s impact on microbial morphology and cell membrane integrity. The antimicrobial efficacy and stability of a topical spray formulation containing stevia extract were evaluated using time-kill and freeze-thaw testing.

**Results:**

The stevia extract yield was 3.59% of the dry plant weight with 259.96 ± 23.66 mg gallic acid equivalent (GAE)/g extract of total phenolics and 247.41 ± 19.92 mg quercetin equivalent (QE)/g extract of total flavonoids. GC-MS analysis identified major volatile components, including N-acetyl-14, 15, 16-trinorlabd-8(17)-en-13-amine (37.70% of peak area), phytol (11.02% of peak area), (-)-spathulenol (9.46% of peak area), n-hexadecanoic acid (8.01% of peak area), and (diphenylphosphinoyloxymethyl) dimethylsilane (7.59% of peak area). The minimum inhibitory concentration of the extract against the tested microorganisms ranged from 0.25 to 128.00 mg/mL. Time-kill kinetics exhibited time- and concentration-dependent germicidal effects. E-SEM and cell leakage analyses indicated that stevia extract compromised microbial cell membrane integrity. A spray formulation containing 10% w/w stevia extract displayed excellent eradication efficacy, achieving a 99.9999% reduction of *S. pseudintermedius* and a 99.999% reduction of *M. pachydermatis* and dermatophytes, with good stability after six freeze-thaw cycles.

**Conclusion:**

Stevia extract is an effective antimicrobial against *S. pseudintermedius*, *M. pachydermatis*, *Mi. canis*, *Mi. gypseum, Mi. gallinae*, and *T. mentagrophytes* *in vitro*. Future research will investigate the pharmaceutical properties and toxicity profiles of purified compounds and determine appropriate dosages and clinical efficacy.

## Introduction

Infectious skin diseases are common dermal issues in animals, and rising antimicrobial resistance among the bacterial, yeast, and dermatophyte species responsible poses substantial challenges in veterinary medicine [1, 2]. The primary cause of pyoderma in companion animals is *Staphylococcus pseudintermedius*, which typically resides in mucosal areas, and it is believed to temporarily inhabit the skin after grooming and licking, particularly in animals experiencing itchiness [3]. Several studies have shown high resistance rates in *S. pseudintermedius* clinical isolates, with 49.3%–85.7% resistance to penicillin, clindamycin, erythromycin, tetracycline, trimethoprim/sulfamethoxazole, and ciprofloxacin and 14%–24% resistance to methicillin [4–6]. *Malassezia* yeast is found on the skin of healthy adult dogs and cats and occasionally in other animals, such as horses, pigs, cows, and goats. *Malassezia pachydermatis* thrives in the interdigital and peri-oral skin regions of healthy animals. Although this yeast is not the most prevalent fungal species, it is linked to conditions that alter skin immune responses and alter chemical and micro-climatic conditions, leading to *Malassezia* dermatitis [7, 8]. Dermatophytes are the primary infectious agents responsible for superficial mycoses, primarily targeting the stratum corneum, nails, and hair. Zoophilic species such as *Microsporum canis*, *Microsporum gallinae*, and *Trichophyton mentagrophytes* typically colonize animals. In addition, geophilic species like *Microsporum gypseum* are present in soil and occasionally lead to infections [9, 10]. *M. pachydermatis* and dermatophyte isolates are increasingly exhibiting reduced susceptibility to commonly used antifungal drugs, including clotrimazole, ketoconazole, miconazole, itraconazole, and terbinafine [11, 12]. In response to this issue, researchers have been investigating herbal extracts as alternative antimicrobial agents in veterinary medicine. These natural compounds have promising antimicrobial activity and offer the added benefit of minimizing the risk of adverse effects commonly associated with synthetic antimicrobial agents [13].

*Stevia rebaudiana* Bertoni (stevia) is a member of the Asteraceae family that is indigenous to South America and is cultivated globally. It is categorized as a perennial plant that can be readily propagated through cuttings, making it a viable candidate for economic cultivation. In particular, it has significant applications in the pharmaceutical and food industries [14]. Although previous studies have shown that stevia extract has powerful antimicrobial properties against several bacterial and fungal species, including *Staphylococcus aureus* [15, 16], *Streptococcus mitis*, *Streptococcus mutans*, *Streptococcus sobrinus* [17], *Epidermophyton* spp., *T. mentagrophytes*, *Candida albicans*, and *Cryptococcus neoformans* [18], limited studies have been conducted on the effectiveness of stevia extract against the bacteria and fungi that cause animal dermatitis.

This study aimed to determine the phytochemical composition of stevia extract and assess its efficacy against six different types of microorganisms commonly causing infectious skin diseases in animals: *S. pseudintermedius*, *M. pachydermatis*, *Mi. canis, Mi. gypseum, Mi. gallinae*, and *T. mentagrophytes*.

## Materials and Methods

### Ethical approval

This study was performed *in vitro* and did not involve any animal subjects. Therefore, ethical approval was not required.

### Study period and location

The study was conducted from October 2023 to September 2024 at the Faculty of Veterinary Medicine, Khon Kaen University, Thailand.

### Stevia extraction

Aerial stevia plant parts were collected from agricultural fields in Khon Kaen Province, Thailand. The plants were botanically identified, and samples were stored in the Khon Kaen University Herbarium (herbarium voucher specimen number: Aiemsaard *et al.*, 02). The collected plants were cleaned with distilled water, dried at 50°C, and crushed into powder. Hexane (Brightchem Sdn Bhd, Malaysia) was used as the extraction solvent through a Soxhlet apparatus at 60°C–70°C for 4 h. The obtained extract was filtered using Whatman filter paper No. 1. The solvent was evaporated using a rotary evaporator (Heidolph, Germany) at 55°C and then stored at 4°C until use [19].

### Total phenolic content

The Folin–Ciocalteu assay was performed according to the method described by Punareewattana *et al*. [19]. Briefly, stevia extract was dissolved in 98% v/v ethyl alcohol (Merck, Germany) to obtain a final concentration of 5% w/v. A 100 μL aliquot of Folin-Ciocalteu reagent (Merck, Germany) was added to 500 μL of stevia extract solution. After standing for 5 min, 100 μL of 20% w/v sodium carbonate (Q RëC, New Zealand) was added, and the final volume was adjusted to 1 mL with 98% v/v ethyl alcohol. The mixture was incubated in the dark for 90 min at room temperature. An ultraviolet (UV)-visible spectrophotometer (Epoch 2, BioTek Instruments, Inc., USA) was used to measure the absorbance at 746 nm. A standard calibration curve was generated using gallic acid (Sigma-Aldrich, Germany) at 25, 50, and 100 μg/mL concentrations. The total phenolic content is expressed as gallic acid equivalents in mg/g of extract (mg GAE/g extract).

### Total flavonoid content

The total flavonoid content of stevia extract was determined by an aluminum chloride colorimetric assay. Briefly, 500 μL of stevia extract solution (5% w/v) was mixed with 50 μL of 0.5% w/v sodium nitrite (Merck, Germany) and allowed to stand for 5 min. Then 50 μL of 10% w/v aluminum chloride was added and left for 5 min. The sample was mixed with 0.5 mL of 1 M sodium hydroxide, and the final volume was adjusted to 1.5 mL with 98% v/v ethyl alcohol. The absorbance at 510 nm was measured, and the total flavonoid content was determined by employing a standard calibration curve of 25, 50, and 100 μg/mL quercetin (Sigma-Aldrich). The results are presented as quercetin equivalents in mg/g of the extract (mg QE/g extract) [19].

### Volatile components

Stevia extract (0.1 g) was dissolved in 2 mL of ethyl acetate. The volatile oil composition of stevia extract was studied by gas chromatography-mass spectrometry (GC-MS) using an Agilent 6890N gas chromatograph and a 5973 Mass Selective Detector (Agilent Technologies, Inc., USA) with a DB-5MS gas chromatography column (5% phenyl 95% dimethyl-poly siloxane fused-silica capillary column; 30 m 0.25 mm, film thickness 0.25 μm). The carrier gas was helium, with a constant flow rate of 1 mL/min. The injection volume (split mode) was 2 μL. The temperature was initially set to 70°C and then raised by 2°C/min until it reached 220°C. The total run time was 85 min, and the mass spectra of the chemical components were compared with mass spectral libraries (Wiley 7n.1, New Jersey, USA) [20].

### Microbial culture

The sources of microorganisms used in the study were: *S. pseudintermedius* American Type Culture Collection (ATCC) 49051, *M. pachydermatis* ATCC 14522, and *Mi. gallinae* ATCC 90749 obtained from the ATCC (Corporate Office, University Boulevard Manassas, Virginia). *Mi. canis* Department of Medical Sciences Thailand (DMST) 29297, *Mi. gypseum* DMST 21146, and *T. mentagrophytes* DMST 19735 were obtained from the DMST (Nonthaburi, Thailand). *S. pseudintermedius* was cultured using Mueller-Hinton broth (MHB) at 37°C for 24 h, whereas *M. pachydermatis* was cultured in Sabouraud dextrose broth (SDB; Becton Dickinson, France) at 37°C for 48 h. Dermatophytes were cultured on Sabouraud dextrose agar (SDA; Becton Dickinson, France) at 37°C for 7 days, phosphate-buffered saline (PBS; pH 7.2) was added, and fungal fragments were collected using a glass spreader. The concentrations of the microbial suspensions were measured using an aerobic plate count assay [20].

### Broth microdilution test

The study was conducted in accordance with the guidelines of the Clinical and Laboratory Standard Institute [21–23], with some adjustments. In brief, the stevia extract was mixed with dimethyl sulfoxide (DMSO; Sigma-Aldrich) and then diluted in MHB (for bacteria) or SDB (for fungi) in 96-well round-bottomed microtiter plates (Corning Incorporated, USA). *S. pseudintermedius* (1 × 10^6^ colony-forming units [CFU]/mL) or fungi (2 × 10^3^ CFU/mL) inocula were added to the wells separately. The microtiter plates containing bacteria and yeast were maintained at 37°C for 24 h, whereas the plates containing dermatophytes were maintained at 30°C for 72 h. Control wells with and without microbial suspensions were used to monitor growth. The minimum inhibitory concentration (MIC) was defined as the lowest concentration of the extract that prevented visible growth after the specified incubation period. The minimum bactericidal concentration (MBC) and minimum fungicidal concentration (MFC) were the lowest concentrations of the extract that prevented the subsequent growth of bacteria on Mueller-Hinton agar (MHA; Becton Dickinson, France) and fungi on SDA. Cephalexin and ketoconazole (Sigma-Aldrich) were used as standard antimicrobial controls.

### Time-kill assay

The time-kill test was conducted following the procedure previously described by Borlace *et al*. [20] with some modifications. Briefly, 900 μL of diluted stevia extract (in PBS) was mixed with 100 μL of inoculum containing 1–5 × 10^7^ CFU/mL of *S. pseudintermedius* and *M. pachydermatis*, and 1 × 10^4^ to 1 × 10^5^ CFU/mL of dermatophytes (final concentrations of stevia extract were 1-, 4-, and 16-times the MIC). The samples were incubated for 1, 2, 4, 6, and 8 h at 30°C, then 10-fold diluted with PBS. Subsequently, 100 μL of the 10^-1^ to 10^-3^ dilutions were spread onto MHA or SDA plates and incubated at 37°C for 24 h (bacteria and yeast) or at 30°C for 72 h (dermatophytes). The number of recovered colonies was recorded, and the outcomes are presented as log_10_ reductions in the number of viable cells. The diluent control contained DMSO (8.88 μL/mL).

### Scanning electron microscopy (SEM)

The effects of stevia extract on microbial morphology were examined using environmental SEM (E-SEM, Thermo Scientific^™^ Quattro-S E-SEM, Thermo Fisher Scientific Inc., USA). Each microbe was treated for 6 h with the extract at a concentration that eradicated at least 99% or 99.9% viable cells based on the time-kill test (except *M. pachydermatis*, which showed a high MIC value: It was tested with 1-times the MIC). The cells were rinsed 3 times with sterile distilled water and centrifuged at 3,000× *g* for 5 min [20]. The resulting sediments were placed on carbon conductive tabs and left to dry in the air before being examined under E-SEM at a magnification ranging from 2,500 to 50,000× in a high vacuum environment at 5–10 kV.

### Leakage study

The microbial suspensions were centrifuged at 3,500× *g* for 10 min. The obtained sediments were washed 3 times with sterile distilled water and resuspended in stevia extract at the same concentration used in the E-SEM study. Microbial cells resuspended in 5% DMSO served as leakage control. Ten minutes before incubation times of 0.5, 1, and 3 h, mixtures were collected and centrifuged at 3,500× *g*. Membrane integrity was determined by measuring the UV absorbance of the supernatant at 260 nm using a UV-visible spectrophotometer (Epoch 2, BioTek Instruments, Inc.). Stevia extract without microbes was used as the blank [20].

### Stevia formulation and stability testing

Stevia extract was developed as a topical spray containing 10% w/w stevia extract, polyethylene glycol 40 hydrogenated castor oil, ethyl alcohol, paraben preservatives, and distilled water. The time-kill test and physical characteristics of the formulation were evaluated before and after undergoing six freeze-thaw cycles lasting 24 h at 5°C followed by 24 h at 40°C. The pH was determined using a pH meter (Lab 850 set pH meter, SI Analytics, Germany), and visual assessment included examination for color changes, sediment presence, and fractionation in the formulations [19, 24].

### Statistical analysis

Each experiment was performed in triplicate. The Shapiro–Wilk test was used to determine data normality. Variances in log_10_ viable cell reduction and pH values pre- and post-freeze-thawing cycles were analyzed using the paired sample t-test for normally distributed data and the Wilcoxon signed-rank test for non-normally distributed data. The statistical analysis was performed using the IBM Statistical Package for the Social Sciences version 28 Software (IBM Corp., NY, USA) with a significance of p ≤ 0.05.

## Results

### Total phenolic and flavonoid contents

The stevia extract was dark-green semi-solid, yielding 3.59% of the dry plant weight. The total phenolic and flavonoid contents are shown in Table-1. Total phenolics were found in 259.96 ± 23.66 mg GAE/g extract and total flavonoids were 247.41 ± 19.92 mg QE/g extract.

**Table-1:** Total phenolic and total flavonoid contents in stevia extract.

Constituent	Amount (per g extract)	Amount (per g dry plant)
Total phenolic	259.96 ± 23.66 mg GAE	10.04 ± 0.85 mg GAE
Total flavonoid	247.41 ± 19.92 mg QE	8.88 ± 0.72 mg QE

Values represent the mean ± SD of triplicate experiments. GAE=gallic acid equivalents, QE=quercitin equivalents

### Volatile component analysis

**Figure-1:**
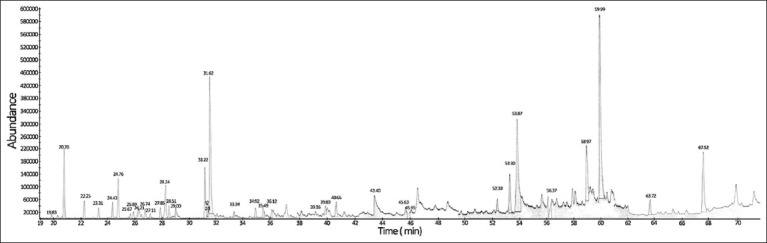
Gas chromatography-mass spectrometry spectrum of volatile oils in stevia extract.

The GC-MS analysis of volatile oils revealed 36 confirmed compounds (Figure-1). N-acetyl-14, 15, 16-trinorlabd-8(17)-en-13-amine was found in the highest proportion (37.70% of peak area), followed by phytol, (-)-spathulenol, n-hexadecanoic acid, and (diphenylphosphinoyloxymethyl)dimethylsilane (11.02%, 9.46%, 8.01%, and 7.59%, respectively). Nerolidol, 1-octadecanol, β-elemene, manoyl oxide, 3-azabicyclo (3.3.1)nonane-7-carboxylic acid, β-farnesene, and δ-cadinene had proportions in the range of 1.58%–3.30%, whereas other substances were <1.00% (Table-2).

**Table-2:** Volatile oil composition of stevia extract from gas chromatography-mass spectrometry analysis.

Compound		% of peak area	Retention time (min)
1	N-acetyl-14, 15, 16-trinorlabd-8 (17)-en-13-amine	37.70	67.52
2	Phytol	11.02	59.99
3	(-)-Spathulenol	9.46	31.62
4	n-Hexadecanoic acid	8.01	53.87
5	(Diphenylphosphinoyloxymethyl) dimethylsilane	7.59	63.72
6	Nerolidol	3.30	31.22
7	1-Octadecanol	3.23	58.97
8	β-elemene	2.75	20.76
9	Manoyl oxide	1.96	53.30
10	3-Azabicyclo (3.3.1) nonane-7-carboxylic acid	1.92	56.37
11	β-Farnesene	1.66	24.76
12	δ-Cadinene	1.58	28.24
13	(+)-δ-Cadinene	0.79	35.49
14	*Trans*-caryophyllene	0.78	22.25
15	7-Acetyl-2-hydroxy-2-mrthyl-5-isopropylbicyclo (4.3.0) nonane	0.70	40.65
16	α-Caryophyllene	0.68	24.34
17	O, O-Diethyl chlorophosphate	0.62	45.95
18	Isospathulenol	0.60	34.92
19	β-Cedren-9	0.57	36.12
20	(-)-Loliolide	0.51	43.40
21	α-Bergamotene	0.50	23.31
22	Bicyclogermacreane	0.47	26.74
23	α-Cedrene	0.47	27.86
24	2, 4-Bis (tert-butyl)-phenol	0.46	28.51
25	Dibutyl phthalate	0.45	52.38
26	Ent-Spathulenol	0.40	39.89
27	Germacrene-D	0.32	25.89
28	Dihydroactinidiolide	0.21	29.00
29	β-Lonone	0.20	26.21
30	β-Cadinene	0.19	27.11
31	Meta-methoxybenzyl alcohol	0.19	33.34
32	γ-Muurolene	0.18	25.67
33	Copaene	0.15	19.83
34	Aromadendrene oxide-(1)	0.14	39.16
35	Epizonaren	0.13	31.45
36	6-Ethyl-1-1, 3-dimethylindan-5-carbaldehyde	0.11	45.63

### Antimicrobial activity of stevia extract

Stevia extract had the same MIC and MBC against *S. pseudintermedius* (1.00 mg/mL) in the broth microdilution test. The MIC and MFC values for the tested yeast and dermatophytes ranged from 0.25 to 128.00 mg/mL (Table-3). *Mi. gallinae* appeared to be more susceptible to stevia extract than the other dermatophytes (MIC was 0.25 mg/mL), followed by *T. mentagrophytes*, *Mi. gypseum, Mi. canis*, with MIC values of 4.00, 8.00, and 16.00 mg/mL, respectively. *M. pachydermatis* was the least sensitive microbe to stevia extract in this study (MIC was 128.00 mg/mL). The MIC of the standard antimicrobial agent cephalexin against *S. pseudintermedius* was 1.00 μg/mL, and the MICs of ketoconazole against yeast and filamentous fungi ranged from 0.078 to 8.00 μg/mL.

**Table-3:** Antimicrobial activity of stevia extract.

	Stevia extract (mg/mL)		Cephalexin (µg/mL)		Ketoconazole (µg/mL)	
		
	MIC	MBC/MFC	MIC	MBC	MIC	MFC
*S. pseudintermedius* ATCC 49051	1.00	1.00	0.125	0.125	NA	NA
*M. pachydermatis* ATCC 14522	128.00	128.00	NA	NA	0.078	0.078
*Mi. canis* DMST 29297	16.00	16.00	NA	NA	4.00	16.00
*Mi. gypseum* DMST 21146	8.00	8.00	NA	NA	8.00	16.00
*Mi. gallinae* ATCC 90749	0.25	0.25	NA	NA	0.50	0.50
*T. mentagrophytes* DMST 19735	4.00	4.00	NA	NA	4.00	8.00

NA=Not applicable. MIC=minimum inhibitory concentration, MBC=minimum bactericidal concentration, and MFC=minimum fungicidal concentration. Values represent mean of triplicate experiments.

### Time-kill kinetics

**Figure-2:**
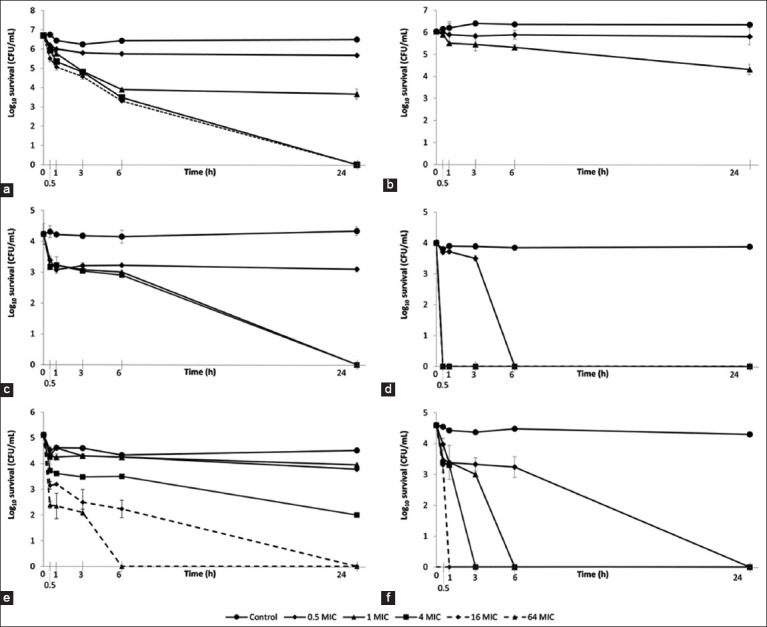
Time-kill kinetics of stevia extract against (a) *Staphylococcus pseudintermedius* American Type Culture Collection (ATCC) 49051 (1 × MIC = 1 mg/mL), (b) *Malassezia pachydermatis* ATCC 14522 (1 × MIC = 128 mg/mL), (c) *Microsporum canis* DMST 29297 (1 × MIC = 16 mg/mL), (d) *Microsporum gypseum* DMST 21146 (1 × MIC = 8 mg/mL), (e) *Microsporum gallinae* ATCC 90749 (1 × MIC = 0.25 mg/mL), and (f) *Trichophyton mentagrophytes* DMST 19735 (1 × MIC = 4 mg/mL). Control: DMSO (8.88 mg/mL).

The time-kill assay results for the stevia extract are presented in Figure-2 and demonstrate concentration- and time-dependent antimicrobial effects. Against *S. pseudintermedius*, the extract achieved <90% (1–log_10_ reduction) eradication at 0.5 and 1 × MIC (0.50 and 1.00 mg/mL) within 30 min to 1 h. However, the antimicrobial efficacy increased as the exposure time or concentration increased. At 1 × MIC, the extract reduced the number of viable bacterial cells by 90% after 3 h, 99% (2-log_10_) after 6 h, and 99.9% (3–log_10_) after 24 h. Higher concentrations of 4 × MIC and 16 × MIC resulted in a 99.9% reduction at 6 h and a 99.9999% (6-log_10_) reduction at 24 h.

The antifungal efficacies of *M. pachydermatis* and dermatophyte species varied. *M. pachydermatis* showed reduced sensitivity to the extract in the time-kill test, with only a 90% reduction in viable cells at 1 × MIC (128.00 mg/mL) after 24 h. In contrast, *Mi. gypseum* was highly susceptible, with >99.99% reduction (4–log_10_) at 0.5 × MIC (4 mg/mL) after 6 h and rapid eradication at 1 × MIC to 16 × MIC (8–64 mg/mL) within 30 min. *Mi. canis* exhibited 90% viability reduction within 30 min and 99.99% after 24 h at concentrations of 1 × MIC to 8 × MIC (16.00–128.00 mg/mL). *Mi. gallinae* showed a 99.999% (5-log_10_) reduction at 16 × MIC (4 mg/mL) after 24 h and at 64 × MIC (16 mg/mL) after 16 h. Finally, the number of viable *T. mentagrophytes* was reduced by 99.99% at 0.5 × MIC, 1 × MIC, 4 × MIC, and 16 × MIC (2.00–64.00 mg/mL) after 1, 3, 6, and 24 h, respectively.

### Leakage analysis and SEM

**Figure-3:**
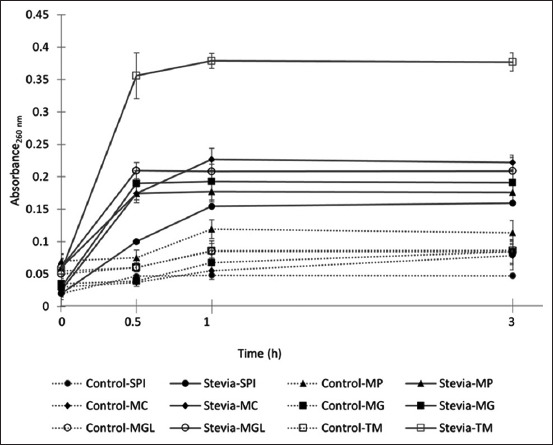
Effect of stevia extract on cell membrane integrity of *Staphylococcus pseudintermedius* American Type Culture Collection (ATCC) 49051 (SPI), *Malassezia pachydermatis* ATCC 14522 (MP), *Microsporum canis* DMST 29297 (MC), *Microsporum gypseum* DMST 21146 (MG), *Microsporum gallinae* ATCC 90749 (MGL), and *Trichophyton mentagrophytes* DMST 19735 (TM). Control: 5% (v/v) DMSO.

**Figure-4:**
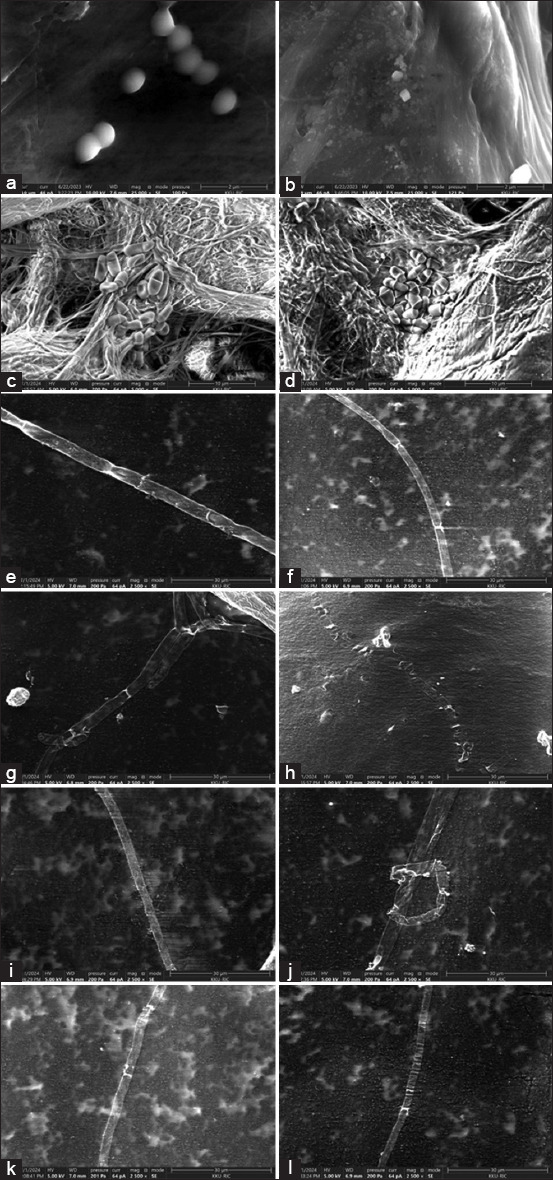
Morphology of microorganisms under environmental scanning electron microscopy after treatment with stevia extract. *Staphylococcus pseudintermedius* American Type Culture Collection (ATCC) 49051: (a) control and (b) treatment; *Malassezia pachydermatis* ATCC 14522: (c) control and (d) treatment (d); *Microsporum canis* DMST 29297: (e) control and (f) treatment; *Microsporum gypseum* DMST 21146: (g) control and (h) treatment; *Microsporum gallinae* ATCC 90749: (i) control and (j) treatment; *Trichophyton mentagrophytes* DMST 19735: (k) control and (l) treatment. Control: 5% (v/v) DMSO.

Stevia extract affected cell membrane integrity, as demonstrated in the leakage study (Figure-3) and caused noticeable alterations in the cellular structure of all the species under E-SEM (Figure-4). The extracellular supernatants of microbial cells treated with stevia extract showed increased absorbance at 260 nm (Abs_260_) compared with the control groups, indicating leakage of intracellular proteins and nucleic acids. The Abs_260_ of the stevia treatment groups ranged from 0.10–0.35 after 30 min, 0.15–0.38 after 1 h, and 0.16–0.38 after 3 h, whereas that of the untreated groups ranged from 0.04–0.08, 0.07–0.12, and 0.08–0.12, respectively. The bacterium *S. pseudintermedius* and the dermatophytes *Mi. gypseum*, *Mi. gallinae*, and *T. mentagrophytes* displayed morphological changes when treated with stevia extract. *S. pseudintermedius* exhibited irregularly shaped fragments and cell debris, in contrast to the small clusters of spheroid cells typical of *Staphylococcus* spp. observed in the untreated control. The untreated dermatophytes showed smooth filaments with well-defined rectangular cells, but after treatment with stevia extract, the filaments appeared rough, with noticeable wrinkles and folds, except for *Mi. canis*, which showed only rough cell walls in some fragments. *M. pachydermatis* cells in the experimental group retained a shape similar to the control group, but the cells became more angular and wrinkled, indicating cell shrinkage.

### Physical properties and antimicrobial efficacy of stevia spray formulations

**Figure-5:**
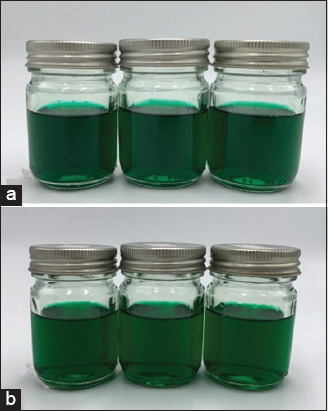
Appearance of the stevia topical spray formulation (a) before and (b) after freeze-thaw test.

**Figure-6:**
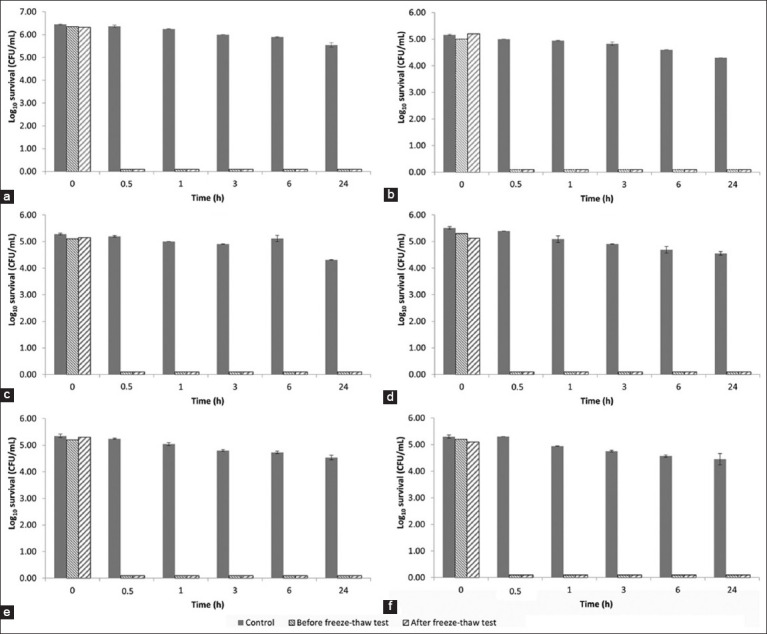
Time-kill kinetics of stevia topical spray formulation against (a) *Staphylococcus pseudintermedius* American Type Culture Collection (ATCC) 49051, (b) *Malassezia pachydermatis* ATCC 14522, (c) *Microsporum canis* DMST 29297, (d) *Microsporum gypseum* DMST 21146, (e) *Microsporum gallinae* ATCC 90749, (f) and *Trichophyton mentagrophytes* DMST 19735. Control: spray formulation base.

The spray formulation containing 10% w/w stevia extract had a dark green color (Figure-5) with the characteristic aroma of stevia and a pH value of 5.01 ± 0.01. After six freeze-thaw cycles, the formulation showed no significant difference in pH value (p > 0.05) or physical properties (Table-4). The time-kill assay conducted before and after freeze-thawing demonstrated potent stevia spray eradication activity against all tested microorganisms (Figure-6). Notably, no viable microbial cells were recovered from the inoculation plates within 30 min of contact time, achieving a 99.9999% reduction of *S. pseudintermedius* and a 99.999% reduction of *M. pachydermatis* and dermatophytes counts. The control spray formulation without stevia extract marginally reduced the number of microorganisms after 24 h (<90% reduction).

**Table-4:** Physical properties of stevia extract spray formulation.

Physical property	Before freeze-thaw test	After freeze-thaw test
pH	5.01 ± 0.01	5.05 ± 0.05
Appearance	Dark-green color with a characteristic aroma of stevia	

## Discussion

This study used hexane, a nonpolar organic solvent, to extract stevia, resulting in an oily semisolid extract. Our results confirmed that the stevia samples were rich in phenolic and flavonoids, which were well dissolved in organic solvents (10.04 ± 0.85 mg GAE and 8.88 ± 0.72 mg QE/g dry plant, respectively). Previous studies [25, 26] have shown that the sample preparation method, extraction method, and solvent type influence the quantity of total phenolics and flavonoids. Toakaenchan *et al*. [26] reported that different drying methods for stevia, including sun drying, shaded drying, hot air oven drying, and microwave-assisted drying, affected the total phenolic and flavonoid contents of an ethanolic extract, with values ranging from 16.75–48.82 mg GAE and 4.39–17.87 mg QE/g dry plant, respectively.

Volatile or essential oils, such as monoterpenes, sesquiterpenes, phenylpropenes, phenols, aldehydes, esters, oxides, amines, amides, ketones, alcohols, nitrogen and sulfur compounds, heterocycles, and their derivatives, are another important class of active phytochemicals in stevia [27]. GC-MS analysis provided qualitative and comparative quantitative data, revealing that N-acetyl-14, 15, 16-trinorlabd-8(17)-en-13-amine, phytol, (diphenylphosphinoyloxymethyl)dimethylsilane, (-)-spathulenol, and n-hexadecanoic acid were the main constituents of the stevia extract. However, GC-MC has limitations, such as its inability to directly analyze the concentration of essential oils in extracts and its reliance on selected spectral libraries, which can result in slight variations in the types of compounds reported in previous studies [28, 29], especially for less studied substances. In addition, the extraction method affects the proportion of volatile oil compositions. Hossain *et al*. [28] identified 62 compounds in stevia’s hydrodistilled essential oil, with α-cadinol (2.98%), caryophyllene oxide (1.23%), (-)-spathulenol (2.21%) and β-guaiene (0.32%) identified as major components. In contrast, Muanda *et al*. [29] reported hydrodistilled oil containing carvacrol (67.89%), caryophyllene oxide (23.5%), spathulenol (15.41%), and cardinol (5.59%) as the major components.

Some secondary metabolites synthesized by plants are recognized for their medicinal properties in traditional and contemporary medicine. Phenolics, flavonoids, and volatile oils represent a substantial proportion of these medicinal compounds and consist of hundreds of substances and their derivatives [30]. Our study revealed a broad range of MIC values for stevia crude extract, showing strong effects against *S. pseudintermedius* and dermatophytes, but limited efficacy against *M. pachydermatis*. Identically, Gamboa and Chaves [17] found that the MIC of stevia extract ranged from 200.00 to 400.00 mg/mL against the bacteria *S. aureus*, *Bacillus cereus*, *Escherichia coli*, and *Klebsiella pneumoniae* and the fungi *Aspergillus flavus*, *Cladosporium herbarum*, *Penicilliumro queforti*, *Trichoderma viride*, *Fusarium chlamydosporum*, and *Macrophomina phaseolina*. Other studies reported MIC values of 3.13–50.00 mg/mL against *S. mutans* [31] and 62.50–500.00 mg/mL against *Lactobacillus acidophilus* [32].

The formulation of the extract as a spray formulation provides excellent microbial eradication efficiency with stable physical and antimicrobial properties. E-SEM and leakage studies revealed significant alterations in the microbial cell structures of all examined pathogens, indicating a potential mechanism for the antibacterial and antifungal properties of stevia extract. The hydroxyl groups found in phenolic and flavonoid compounds and in some volatile components may bind to microbial cell membranes and disrupt their structure. This disruption affects various cellular functions, leading to membrane expansion, increased permeability, interference with membrane proteins, and alterations in ion transport mechanisms [33–35]. Along with their primary antimicrobial effects, phenolic and flavonoid compounds also possess significant antioxidant qualities that may enhance their effectiveness in treating dermatitis. Antioxidants help lower oxidative stress by neutralizing reactive oxygen species, which can consequently alleviate or avert the inflammatory processes that worsen dermatitis lesions [36, 37].

The stevia extract formula developed in this research is an aqueous solution containing ethyl alcohol as the primary solvent and polyethylene glycol 40 hydrogenated castor oil as a non-ionic surfactant and solubilizing agent with moisturizing benefits. This formulation may improve the extract’s penetration through the epidermis, thereby increasing its antimicrobial effectiveness [38]. Consequently, it could serve as either an alternative treatment or be used alongside antimicrobial medications to manage skin infections in animals, potentially decreasing antibiotic reliance.

## Conclusion

This study highlights the potential of stevia extract as a promising antimicrobial agent for addressing a broad spectrum of pathogens responsible for skin infections in animals, including both bacterial and fungal species. The demonstrated antibacterial and antifungal activities, coupled with insights into the phytochemical composition and possible mechanisms of action, underline the extract’s therapeutic potential. However, the study’s limitations should be noted, particularly the absence of testing on microbial strains isolated from animal lesions and the lack of *in vivo* evaluations to confirm efficacy and safety in clinical settings. Future research should focus on isolating and characterizing the active compounds from the crude extract, conducting detailed toxicity studies, and determining appropriate dosages. Additionally, clinical trials on affected animals are crucial to validate the extract’s pharmaceutical efficacy and practical application as an antimicrobial spray formulation.

## Authors’ Contributions

RS and PS: Prepared the plant samples, performed stevia extraction, and contributed to antimicrobial testing. GNB: Prepared the stevia spray formulation, tested the stability, and drafted and revised the manuscript. ET: Determined total phenolic, total flavonoid, and volatile oil contents in the extract and drafted and revised the manuscript. JA: Antimicrobial testing, SEM, leakage study, and drafted the manuscript. All authors have read and approved the final manuscript.
